# Excess mortality for care home residents during the first 23 weeks of the COVID-19 pandemic in England: a national cohort study

**DOI:** 10.1186/s12916-021-01945-2

**Published:** 2021-03-05

**Authors:** Marcello Morciano, Jonathan Stokes, Evangelos Kontopantelis, Ian Hall, Alex J. Turner

**Affiliations:** 1 Health Organisation, Policy and Economics (HOPE) Research Group, University of Manchester, Manchester, M13 9PL UK; 2 NIHR School for Primary Care Research, University of Manchester, Manchester, M13 9PL UK; 3 Division of Informatics, Imaging and Data Sciences, University of Manchester, Manchester, M13 9PL UK; 4 Department of Mathematics, University of Manchester, Manchester, M13 9PL UK

**Keywords:** Care homes, COVID-19, Excess deaths, England

## Abstract

**Background:**

To estimate excess mortality for care home residents during the COVID-19 pandemic in England, exploring associations with care home characteristics.

**Methods:**

Daily number of deaths in all residential and nursing homes in England notified to the Care Quality Commission (CQC) from 1 January 2017 to 7 August 2020. Care home-level data linked with CQC care home register to identify home characteristics: client type (over 65s/children and adults), ownership status (for-profit/not-for-profit; branded/independent) and size (small/medium/large). Excess deaths computed as the difference between observed and predicted deaths using local authority fixed-effect Poisson regressions on pre-pandemic data. Fixed-effect logistic regressions were used to model odds of experiencing COVID-19 suspected/confirmed deaths.

**Results:**

Up to 7 August 2020, there were 29,542 (95% CI 25,176 to 33,908) excess deaths in all care homes. Excess deaths represented 6.5% (95% CI 5.5 to 7.4%) of all care home beds, higher in nursing (8.4%) than residential (4.6%) homes. 64.7% (95% CI 56.4 to 76.0%) of the excess deaths were confirmed/suspected COVID-19. Almost all excess deaths were recorded in the quarter (27.4%) of homes with any COVID-19 fatalities. The odds of experiencing COVID-19 attributable deaths were higher in homes providing nursing services (OR 1.8, 95% CI 1.6 to 2.0), to older people and/or with dementia (OR 5.5, 95% CI 4.4 to 6.8), amongst larger (vs. small) homes (OR 13.3, 95% CI 11.5 to 15.4) and belonging to a large provider/brand (OR 1.2, 95% CI 1.1 to 1.3). There was no significant association with for-profit status of providers.

**Conclusions:**

To limit excess mortality, policy should be targeted at care homes to minimise the risk of ingress of disease and limit subsequent transmission. Our findings provide specific characteristic targets for further research on mechanisms and policy priority.

**Supplementary Information:**

The online version contains supplementary material available at 10.1186/s12916-021-01945-2.

## Background

Globally, residents in care homes have experienced disproportionately high morbidity and mortality from COVID-19. Across Europe, countries adopting more relaxed strategies to tackle the pandemic, such as Sweden, and those adopting severe lockdowns, like Spain and the UK, have both struggled to protect vulnerable persons in care homes [1, 2]. Early international evidence suggests that nearly half of all COVID-19 deaths in five European countries were amongst care home residents [2].

Directly attributed COVID-19 deaths do not necessarily capture the full impact on mortality, however [3]. The death toll for COVID-19 relies on SARS-CoV-2 testing, with tests particularly supply-constrained in early parts of the pandemic. Indirect fatalities due to non-COVID-19-related causes might also have increased. For example, through increased risks of harm from isolation [4] and possible delayed/cancelled hospital admissions resulting in unintended iatrogenic events and further deaths. Excess deaths, the additional deaths observed in a given period compared to the number usually expected, better capture direct and indirect mortality impacts.

The Office for National Statistics (ONS) in England and Wales have reported aggregated excess deaths by place of occurrence. There was approximately a 79% increase in total deaths in care homes in England and Wales from 2 March to 12 June compared to 2015–2019 [5, 6]. These aggregates, however, do not account for care home residents dying in other settings (e.g. hospital), nor provide sufficient information to reflect on the impacts of enacted policies over the period, or to inform new policies for future virus waves.

Likely, not all care homes suffered equally from COVID-19 [7]. In Canada, for-profit status was associated with the number of residents infected and deaths after an outbreak [8]. Nursing homes with higher nurse staffing hours per resident were less likely to experience outbreaks across eight US states [9]. In 179 UK care homes, lower infection rates were found in small homes with high staff-to-resident ratios and low bed occupancy rates [10]. Large care homes experienced higher rates of infection in Wales [11], and in the Lothian region of Scotland, excess deaths were concentrated in a minority of care homes with an outbreak [12]. A national breakdown of excess deaths by care home characteristics is largely lacking from the current literature in England [6].

The aim of this study is to use nationally representative administrative data from all care homes in England to quantify the excess mortality for residents during the first 23 weeks of the COVID-19 outbreak, and to explore associations with care home characteristics. This analysis highlighted that almost all excess deaths for care home residents in England were recorded in homes with any COVID-19 fatalities. We therefore complement the analysis by using multivariable logistic regressions to estimate the odds of care homes experiencing a suspected or confirmed COVID-19 death across care home types. This knowledge might inform a targeted policy response in future waves.

## Methods

### Institutional context

The English care home market is mostly private and very fragmented, with the type of service provided, client types covered and bed capacity varying systematically by provider-type and the local authorities in which they operate [13].

There are two main categories of care homes: care homes providing nursing services and those which do not. Care homes which do provide nursing services (nursing homes) cater for people who have complex clinical needs that require regular attention from registered nurses. Care homes which do not offer nursing care (referred to as residential homes) cater for people who often require personal care only, with district nurses and physicians called in when necessary. Residential care homes differ from assisted living facilities which are not included in this analysis. Similar to assisted living, residential homes provide personal care such as washing and dressing, but unlike assisted living, residential homes provide full-time accommodation in a group setting.

Further differences in care homes’ organisational characteristics and operational strategies (for-profit/not-for-profit, independent or belonging to a corporate chain/groups of providers (branded), small/medium/large) might also influence the ability of care homes to put in place effective infection, prevention and control protocols, for example, advanced care planning to ensure patient-centred management, ability to access personal protective equipment (PPE), SARS-CoV-2 testing capacity constraints and staff-to-resident ratios and policies on staff and patient movement across facilities [14].

Policies adopted in the wider health and care system might have also impacted COVID-19 infections in English care homes. In mid-March, hospital trusts discharged medically fit patients to care homes to free capacity [15]. Mandatory testing prior to discharge was only brought into effect a month later [16, 17]. On March 24, the wider population were ordered not to leave their home except for “essential” reasons [18], including visiting care homes, later clarified to only allowed in exceptional circumstances such as at the end of life.

### Data

Care home-level daily death notification data sent by registered care home operators in England in the period 1 January 2017 to 7 August 2020 to the Care Quality Commission (CQC), the independent regulator of health and adult social care in England. All providers must send their notifications to CQC without delay and “are typically provided within two to three days of death” [19]. These have been validated against ONS data, showing a high level of accuracy [20].

The data includes all deaths of care home residents regardless of whether they occurred in care homes or elsewhere (e.g. in hospital) [21]. From 10 April 2020, deaths suspected (based on the statement of care home providers) or confirmed (tested) to be attributable to COVID-19 (COVID-19 deaths) were also identified [20]. All other deaths are classified as non-COVID-19 deaths.

Death notification data were linked at a care home-level with CQC registers of active care homes in England, providing data on care home characteristics: setting type (nursing or residential home), client types (offering services for people aged 65+ and/or people with dementia or offering services to children and adults), ownership status (whether not-for-profit—charity/NHS/local-authority-run homes—or for-profit), whether known to CQC to be independent or affiliated to a large provider/brand and their registered maximum bed capacity (coded as small (less than 23 beds), medium (24–40 beds) and large (41 or more beds), see Additional file 1: Appendix 1 for a justification of the chosen cut-offs).

Providers enter and leave the market over time. In January 2017, there were 16,481 active care homes, reducing to 15,554 by January 2020. Bed capacity is more stable, with 460,323 beds in January 2017 and 457,347 in January 2020. For the calculation of excess deaths, we use data from the 13,630 care homes which reported at least one death over the study period. Thirty-two (0.17%) care homes were excluded from the analysis due to the inability to match with CQC registers. Of all 19,271 care homes reported to be active at some point between 1 January 2017 and 7 August 2020, 3747 care homes were no longer active in March 2020 (de-activated providers). These were made up of care homes which are known to have closed their activity (53%) and those who experienced a change in their legal status (47%). The leading cause for the latter group is a change of ownership (87%). Other main causes pertain to a change in their legal entity (8%) or moved location (4%). When modelling the odds of experiencing any COVID-19 fatalities, we have focused on active providers in March 2020 (15,524 care home) and have therefore excluded de-activated providers.

To calculate excess deaths overall and by care home type, we aggregated daily care home-level deaths to weekly and local authority level. This aimed to reduce the incidence of zeros and the non-constant intra-week variation in death counts (Additional file 1: Appendix 1, p.1). Therefore, excess deaths were estimated using aggregated data for 150 local authorities for a period of 188 weeks: 165 weeks (1 January 2017 to 3 March 2020) as the pre-COVID-19 period and 23 weeks (4 March 2020 to 7 August 2020) as the post-COVID-19 period, with the start of the COVID-19 period defined by the first week in which one COVID-19 death was reported in England [22].

### Methods

To calculate excess deaths overall and by care home characteristics, we first used data from the pre-COVID-19 period to estimate expected death trends [23]. After comparing predictive accuracy with more complex models structures (Additional file 1: Appendix 2,p.3), a Poisson regression model (standard log link) was selected, with covariates including a quartic polynomial of week-of-the-year (to account for seasonality) and local authority fixed effects (to account for determinants of deaths that differ across local authorities but do not change over time). We also examined the robustness of excess death estimates to these alternative approaches (Additional file 1: Appendix Table A2-4). Predicted weekly deaths for each local authority were used as the estimated counterfactual in the COVID-19 period (i.e. the deaths that would have occurred in the absence of the pandemic). National excess deaths were computed as the sum of the difference between observed and counterfactual deaths over all local authority weeks. Excess deaths per hundred bed capacity (excess deaths per bed) were also reported. 95% confidence intervals were constructed by bootstrapping with local authority resampling (50 replications). We also report observed weekly deaths flagged as confirmed or suspected COVID-19 fatalities to show the proportion of excess deaths directly attributed to COVID.

The timing and pattern of excess deaths were likely to vary considerably according to whether a care home has experienced an outbreak or not. As nationwide care home-level data on COVID-19 outbreaks are collated but not publicly available [24], we classified care homes according to whether a confirmed/suspected COVID-19 death had been reported in the 23-week COVID-19 period. We used univariate logistic regression to estimate unadjusted associations between each care home characteristic and odds of reporting any COVID-19 fatalities. Adjusted odds were computed using multivariable models including all care home characteristics as covariates, as well as local authority fixed effects to control for all time-invariant area differences across local authorities (including unobservable determinants of disease spread at the area level). This analysis accounts for the likely collinearity amongst categories (e.g. almost all nursing homes cater for older people with dementia and are of medium/large size), bringing additional adjustments for multiple confounders simultaneously, which is not possible when estimating excess deaths alone. Covariate selection was determined by the availability of data on care home characteristics from the CQC. To further explore the potential impact of local demographics, with robustness checks, we further tested the impact of including dummy variables on whether the care home is located in (a) a postcode flagged as “urban” and (b) the least and most deprived 20% of lower super output areas in England according to the 2019 Index of Multiple Deprivation.

All analyses were performed in StataMP v14·2.

## Results

### Descriptive statistics

Table 1 summarises the characteristics of all care homes in England active in March 2020. The minority (4428, 28.5%) provided nursing services. However, on average, nursing homes had more beds than residential homes (50.6 beds versus 20.9) since they are larger (60.2% versus 12.9% with 41+ beds). This explains the more comparable supply of total beds in nursing (223,917) and residential care homes (231,677).

**Table 1:** Characteristics of the care homes in England

	Overall (nursing and residential homes combined)	Nursing homes	Residential homes
Care homes	15,524	4428	11,096
Average bed capacity	29.3	50.6	20.9
Total bed capacity offered	455,594	223,917	231,677
Care home size			
Small homes [0–23 beds]	49.2%	12.0%	64.1%
Medium homes [24–40 beds]	24.4%	27.8%	23.0%
Large homes [41+ beds]	26.4%	60.2%	12.9%
Service type			
Providing services to older people and/or people with dementia	71.7%	93.4%	63.1%
Providing non-dementia services to children and/or adults only	28.3%	6.6%	36.9%
Provider type			
Branded (chain ownership)	37.7%	45.8%	34.5%
Non-branded (independent ownership)	62.3%	54.2%	65.5%
Legal status			
For-profit	85.3%	90.5%	83.3%
Not-for-profit	14.7%	9.5%	16.7%
% care homes reporting any death in the COVID-19 period	59.7%	89.0%	47.9%
% care homes reporting confirmed/suspected COVID-19 fatalities	27.4%	54.2%	16.7%

Own elaboration on CQC data on care homes reported to be active in March 2020

Almost all (93.4%) nursing homes in England provided services to older people and/or people with dementia, as well as the majority (63.1%) of residential homes.

Over a third (37.7%) of care homes were affiliated to a larger branded provider/chain. The proportion of branded care homes was higher (45.8%) for nursing care homes than for residential homes (34.5%). 90.5% of nursing homes were for-profit, with slightly less (83.3%) residential homes.

Overall, approximately 6 in 10 care homes experienced at least one death in the COVID-19 period, with a larger share of nursing homes (89%) reporting fatalities than residential homes (47.9%). A total of 5641 care homes were active at some point in the study period but did not experience fatalities. These were mainly small residential homes, with an average bed capacity of 9.21 (95% CI 8.95 to 9.49). 27.4% of care homes reported COVID-19-confirmed/suspected fatalities, most in nursing (54.2%) rather than residential homes (16.7%).

### Observed, expected and excess deaths

**Fig. 1:**
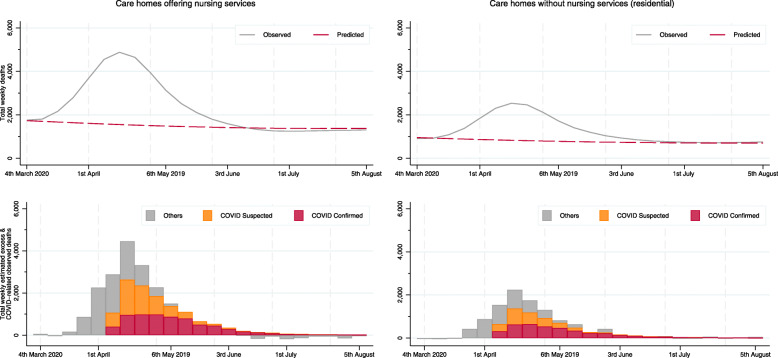
Predicted versus observed deaths, and estimated excess deaths by care home setting type in the first 23 weeks of the COVID-19 pandemic in England

Prior to the COVID-19 period, predicted deaths tracked observed deaths relatively closely (Additional file 1: Appendix 3, p.9). During the COVID-19 period, observed deaths were considerably higher than predicted from historical trends (Fig. 1). Most excess deaths occurred during the 10 weeks between 25 March and 2 June. At the end of the study period, excess deaths were lower than predicted, especially in nursing homes. Overall, 64.7% of calculated excess deaths (95% CI 56.4 to 76.0%) were reported to be attributable to confirmed/suspected COVID-19, with this proportion increasing over time.

There were 29,542 (95% CI 25,176 to 33,908) excess deaths in all care homes over the COVID-19 period (Table 2), equivalent to excess deaths per bed of 6.3% (95% CI 5.4 to 7.2%). Robustness checks (Additional file 1: Appendix 2, p.8) showed consistent excess death estimates across different modelling approaches (range 29,542 to 29,711).

**Table 2:** Excess deaths (panel a) and excess deaths per bed (panel b) by care home type (in the first 24 weeks with COVID-19)

	Overall (nursing and residential homes combined)	Nursing homes	Residential homes
Panel a. Total excess deaths			
Overall excess death	29,542 [25,176; 33,908]	18,891 [15,956; 21,826]	10,651 [8914; 12,388]
*% excess deaths attributable to COVID-19*	64.7 [56.41; 75.98]	67.1 [58.11; 79.49]	60.5 [52.03; 72.30]
Reporting COVID-19 deaths	29,429 [25,047; 33,810]	19,722 [16,665; 22,778]	9707 [8011; 11,403]
Not reported	112 [−457; 682]	− 831 [− 1291; −371]	943 [503; 1383]
Providing services to older people/with dementia	28,958 [24,673; 33,242]	18,786 [15,858; 21,714]	10,171 [8470; 11,873]
Providing non-dementia services to children and/or adults only	584 [380; 789]	105 [44; 166]	479 [292; 666]
For-profit care homes	25,468 [21,685; 29,251]	17,335 [14,782; 19,888]	8133 [6592; 9674]
Not-for-profit care homes	4074 [3081; 5067]	1556 [858; 2255]	2517 [2005; 3030]
Branded care homes	14,671 [12,053; 17,288]	9776 [8017; 11,534]	4895 [3829; 5962]
Non-branded care homes	14,871 [12,702; 17,041]	9116 [7540; 10,692]	5755 [4864; 6647]
Small homes [0–23 beds]	1752 [1410; 2095]	195 [55; 335]	1558 [1231; 1885]
Medium homes [24–40 beds]	5751 [4791; 6711]	2480 [1869; 3092]	3270 [2770; 3771]
Large homes [41+ beds]	22,039 [18,564; 25,513]	16,216 [13,704; 18,729]	5823 [4524; 7122]
Panel b. Total excess deaths per bed (in %, adjusted for bed capacity as reported in March 2020)			
Overall excess	6.5 [5.5; 7.4]	8.4 [7.1; 9.7]	4.6 [3.8; 5.3]
*Overall excess attributable to COVID-19*	4.2 [3.1; 5.7]	5.7 [4.1; 7.7]	2.8 [2.0; 3.9]
Reporting COVID-19 deaths	13.5 [11.5; 15.5]	13.8 [11.7; 15.9]	13.0 [10.7; 15.2]
Not reported	0.0 [−0.2; 0.3]	−1.0 [−1.6; −0.5]	0.6 [0.3; 0.9]
Providing services to older people/with dementia	7.0 [5.9; 8.0]	8.6 [7.3; 9.9]	5.1 [4.3; 6.0]
Providing non-dementia services to children and/or adults only	1.5 [1.0; 2.0]	1.9 [0.8; 3.0]	1.4 [0.9; 2.0]
For-profit care homes	6.3 [5.4; 7.2]	8.4 [7.2; 9.7]	4.1 [3.3; 4.9]
Not-for-profit care homes	8.0 [6.1; 10.0]	8.7 [4.8; 12.6]	7.7 [6.1; 9.2]
Branded care homes	7.2 [6.0; 8.5]	8.4 [6.9; 10.0]	5.6 [4.4; 6.9]
Non-branded care homes	5.8 [5.0; 6.7]	8.4 [7.0; 9.9]	4.0 [3.4; 4.6]
Small homes [0–23 beds]	2.2 [1.8; 2.7]	2.6 [0.7; 4.5]	2.2 [1.7; 2.6]
Medium homes [24–40 beds]	4.7 [4.0; 5.5]	6.0 [4.6; 7.5]	4.1 [3.5; 4.7]
Large homes [41+ beds]	8.6 [7.3; 10.0]	9.2 [7.8; 10.7]	7.3 [5.6; 8.9]

Excess deaths were higher in nursing (18,891, 95% CI 15,956 to 21,826) compared to residential (10,651, 95% CI 8914 to 12,388) homes, with almost double excess deaths per bed (8.4% versus 4.6%).

Excess deaths were significantly higher in facilities that provided services to older people and people with dementia, mainly for nursing homes, with excess deaths per bed of 8.6% (95% CI 7.3 to 9.9%).

For-profit and not-for-profit nursing homes had comparable excess deaths per bed, but the rate was nearly double in not-for-profit residential homes (7.7% versus 4.1%).

Branded homes experienced higher excess deaths per bed than independent care homes (7.2% versus 5.8%). This difference largely occurred amongst residential homes (5.6% versus 4%).

Larger care homes also had higher excess deaths per bed (8.6% versus 2.2% in small homes).

The starkest difference in excess deaths was between care homes that experienced and did not experience at least one suspected/confirmed COVID-19 death, with the former responsible for almost all excess deaths (29,429, 95% CI 25,047 to 33,810). For graphical inspection, see Additional file 1: Appendix 4, p.12. For homes experiencing COVID-19-attributable fatalities, excess deaths per bed were comparable across settings (13.8% nursing; 13.0% residential). Estimated excess deaths for nursing homes not reporting COVID-19 deaths were negative (− 831, 95% CI − 1291 to − 371), corresponding to estimated excess deaths per bed of − 1% (95% CI − 1.6 to − 0.5%). On the other hand, there were 943 excess deaths (95% CI 503 to 1383) in residential homes not reporting COVID-19 deaths, corresponding to excess deaths per bed of 0.6% (95% CI 0.3 to 0.9%).

### Care home characteristics associated with odds of one COVID-19 death

After adjusting for other care home characteristics, nursing homes had a statistically significant higher odds of experiencing COVID-19-confirmed/suspected deaths than residential homes (OR 1.8, 95% CI 1.6 to 2.0) (Table 3). Care homes offering services to older people and/or people with dementia had higher odds of a COVID-19 death than homes providing non-dementia services to children and/or adults only (OR 5.5, 95% CI 4.4 to 6.8). Branded care homes experienced significantly higher odds of one COVID-19-related death compared to independent homes (OR 1.2, 95% CI 1.1 to 1.3), although we find no evidence of increased odds associated with for-profit status (overall OR 1.0, 95% CI 0.8 to 1.1). Compared to small care homes, medium-sized facilities experienced higher odds of COVID-19-related deaths (OR 5.2, 95% CI 4.5 to 6.0), with large-sized homes experienced even greater odds (OR 13.3, 95% CI 11.5 to 15.4). Results were robust to controlling for deprivation and urbanicity of the care home location and to restricting the sample to care homes providing services to older people/with dementia (Additional file 1: Appendix 5, p.15).

**Table 3:** The odds ratios (and 95% CI) of experiencing COVID-19-confirmed/suspected deaths in the English care homes

	% of care homes in each category reporting COVID-19 deaths	Unadjusted OR (95% CI)			Multivariable adjusted OR (95% CI)		
		OR	95% CI	*p* value	OR	95% CI	*p* value
Overall (nursing and residential combined)							
Providing residential services only	14.9	1	(Reference)		1	(Reference)	
Providing nursing services	20.0	5.93	(5.47–6.42)	< 0.001	1.81	(1.64–1.99)	< 0.001
Providing non-dementia services to children and/or adults only	19.2	1	(Reference)		1	(Reference)	
Providing services to older people/with dementia	17.0	26.87	(21.95–32.88)	< 0.001	5.45	(4.36–6.81)	< 0.001
For-profit care homes	16.6	1	(Reference)		1	(Reference)	
Not-for-profit care home	21.2	0.54	(0.48–0.61)	< 0.001	0.96	(0.83–1.11)	0.605
Non-branded care homes	14.4	1	(Reference)		1	(Reference)	
Branded care homes	21.7	1.6	(1.48–1.72)	< 0.001	1.21	(1.1–1.34)	< 0.001
Small homes [0–23 beds]	11.4	1	(Reference)		1	(Reference)	
Medium homes [24–40 beds]	14.7	10.84	(9.53–12.33)	< 0.001	5.2	(4.52–5.98)	< 0.001
Large homes [41+ beds]	21.9	35.69	(31.43–40.52)	< 0.001	13.27	(11.45–15.37)	< 0.001
Nursing							
Providing non-dementia services to children and/or adults only	22.7	1	(Reference)		1	(Reference)	
Providing services to older people/with dementia	20.0	9.99	(6.93–14.42)	< 0.001	2.98	(1.98–4.49)	< 0.001
For-profit care homes	19.6	1	(Reference)		1	(Reference)	
Not-for-profit care home	24.8	0.59	(0.47–0.73)	< 0.001	0.91	(0.71–1.17)	0.48
Non-branded care homes	17.4	1	(Reference)		1	(Reference)	
Branded care homes	23.1	1.61	(1.42–1.83)	< 0.001	1.26	(1.1–1.45)	0.001
Small homes [0–23 beds]	14.5	1	(Reference)		1	(Reference)	
Medium homes [24–40 beds]	16.3	5.97	(4.43–8.03)	< 0.001	4.39	(3.21–5.99)	< 0.001
Large homes [41+ beds]	22.1	16.10	(12.1–21.43)	< 0.001	10.88	(8.03–14.74)	< 0.001
Residential							
Providing non-dementia services to children and/or adults only	18.2	1	(Reference)		1	(Reference)	
Providing services to older people/with dementia	14.7	21.8	(16.97–28)	< 0.001	6.57	(5–8.63)	< 0.001
For-profit care homes	14.3	1	(Reference)		1	(Reference)	
Not-for-profit care home	19.5	0.73	(0.63–0.85)	< 0.001	0.99	(0.83–1.19)	0.943
Non-branded care homes	12.8	1	(Reference)		1	(Reference)	
Branded care homes	20.2	1.31	(1.17–1.46)	< 0.001	1.16	(1.02–1.33)	0.028
Small homes [0–23 beds]	10.9	1	(Reference)		1	(Reference)	
Medium homes [24–40 beds]	14.0	9.70	(8.34–11.27)	< 0.001	5.12	(4.36–6.00)	< 0.001
Large homes [41+ beds]	21.5	27.50	(23.35–32.40)	< 0.001	13.45	(11.26–16.08)	< 0.001

A total of 15,524 care homes in England (4428 nursing and 11,096 residential) reported to be active in March 2020 to CQC. Adjusted odds computed using multivariable models with local authority-fixed effects

## Discussion

### Principal findings

Using provider-level administrative data on all care homes in England, we estimated that there were over 29,500 excess deaths of care home residents during the first 23 weeks of COVID-19, equivalent to 6.5% of all care home beds. Almost 65% of the excess deaths were reported to be directly attributable (confirmed/suspected) to COVID-19. Our analysis shows that almost all excess deaths were recorded in the quarter of care homes which reported COVID-19 fatalities. This highlights that (i) non-COVID-19-attributed excess deaths were likely to be directly due to COVID-19 and/or (ii) any indirect negative effects of COVID-19 and enacted policies on mortality were predominantly constrained to those homes experiencing an outbreak. Non-COVID-19-attributed deaths being reported mainly during the early stages of the pandemic, when CQC recording of COVID-19 death was missing (before 10th April), guidance focused on a narrower set of symptoms and there was a shortage of testing, providing support for the former hypothesis.

Excess deaths were mainly concentrated amongst large and branded homes that provide services to older people and people with dementia. Adjusted care home level analysis confirmed these findings.

### Strengths and limitations

To our knowledge, this is the first independent analysis that uses national administrative records from all care homes in England to estimate the impact of COVID-19. We find comparable total deaths to official estimates [5], adding stratifications of excess deaths by key care home characteristics and multivariable analysis to add a more nuanced understanding of these deaths. Local authority fixed effects were used to account for time-invariant measured and unmeasured determinants and confounders that differ across the local authority.

Our study also has limitations. Firstly, due to a high incidence of zeros at the individual care home level, it is not reliable to calculate the number of excess deaths per care home. Instead, we aggregated excess deaths to the local authority level and stratify by univariate care home characteristics in turn. To incorporate multivariable analysis with care home characteristics, we instead estimate odds of COVID-19 care home deaths as a proxy for odds of excess deaths. The univariate analysis suggests this should be a good proxy since almost all excess deaths occur in a care home with at least one recorded COVID-19 death.

We can observe the counts of COVID-19-attributed fatalities across care homes but not whether non-fatal COVID-19 cases occurred. This case data is not available, though serological and whole-genome sequencing studies give insights into this [24]. The attribution of COVID-19-related deaths is based on statements from providers to the CQC starting from 10 April 2020 and not always testing-confirmed or reflected in the death certificate. COVID-19-attributable deaths that occurred before 10 April would have been miscoded. The reported lower rates of testing could lead to some relevant deaths not having COVID-19 listed as a contributory factor, leading to apparently higher non-COVID-19 excess deaths [5, 10, 20].

No data was available on occupancy rates at the care home level. We instead used maximum bed capacity as reported in March 2020, assuming full occupancy. In the UK, occupancy rates were estimated to be on average 90% in nursing homes and 91% in residential homes [13]. It is very likely that occupancy rates declined during the COVID-19 period. However, assuming an arbitrary lower occupancy would increase excess mortality rates only proportionally, unless further breakdowns by time and care home types became available.

Measures of staffing and working conditions, and individual care home shortages of equipment would have been relevant for this analysis but there is no national care home-level data available. We also lacked data on residents’ case mix and their socio-demographic status. Our analysis is instead based on providers’ characteristics as reported to CQC. However, arguably, providers, rather than individual patients, are the targets of policy intervention and therefore these are the most relevant to include.

This study used administrative data and so sample sizes were not under our control. Despite the relatively large sample size available for examining the associations between the odds of one care home death and care home characteristics (> 15,000), it is possible that our analysis may not be powered to detect statistically significant associations with some characteristics, such as for-profit status.

We did not account for exposure and incidence of COVID-19 in the local area where each care home is located, or local policy responses to the pandemic, which changed over time. Wider community testing was negligible in the early parts of our analysis period [25] and likely differed by local authority capacity which would bias results if included. Furthermore, staff, healthcare professionals and any other individuals entering the care homes are not necessarily from or have only interacted with the immediate local areas [26], so fully capturing this would require location data for multiple individuals over time. Good-quality data on local policy responses was also unavailable.

Finally, as the number of deaths in the absence of the outbreak cannot be observed but only predicted, there is the potential that market dynamics and prediction errors could have influenced excess deaths estimates. However, we estimated small prediction errors in the pre-COVID-19 period relative to the size of excess deaths in the COVID period. Excess death estimates were also robust to different modelling approaches.

### Study in context

By comparing observed deaths against averages over a historical 5-year period, the ONS estimated 25,876 excess deaths in English care homes up to 8 August [5]. Our estimates exceed this slightly. In addition to differences in methods, this is likely due to our data including deaths of care home residents occurring outside of a care home setting (e.g. in hospital).

Consistent with previous studies, we find that excess deaths occur overwhelmingly in the minority of care homes that experience COVID-19 fatalities [12]. This might suggest higher proportions of COVID-19-related excess deaths than reported [27] and that some deaths are potentially avoidable if initial care home outbreaks had been prevented. Although national lockdowns have the potential to displace care for care home residents with health conditions other than COVID-19, coupled with evidence of increases in mood and behavioural problems [28], our finding of no excess deaths in care homes without care home fatalities suggests that these issues may not impact mortality in the short run. Whether this type of excess mortality emerges in the longer-term in a subject for future research.

However, our results suggest that other care home characteristics, relating to the type of residents, staffing, ownership and size, are also important.

Care homes providing services to older people/with dementia suffered most deaths. This is unsurprising given the increased risk of contracting SARS-CoV-2 (difficulties complying with physical distancing, masking and hand hygiene) and increased risk of morbidity/mortality (comorbid illnesses), frailty and age. However, for care homes serving this group, there were smaller odds of COVID-19-related deaths in nursing compared with residential care homes. This might suggest a protective effect of the presence of staff with nursing backgrounds and infection, prevention and control (IPC) training, as found in other settings [9].

Overall, though, nursing homes had the most excess deaths and odds of COVID-19-confirmed/suspected deaths. This is likely due to these homes containing residents at high risk of contracting and dying of SARS-CoV-2, increased frailty and higher prevalence of co-morbidities, and therefore a greater likelihood of being in contact with other healthcare settings and practitioners [29].

In line with the existing literature, we found that large care homes are more likely to experience negative outcomes [10–12]. A likely contributor is that larger homes have a higher footfall altogether, of staff, healthcare workers, residents flowing in and out of hospitals, and visitors in non-pandemic times. This increases their chances of exposure to an infected individual, particularly in the absence of rigorous testing. Furthermore, it might be easier to ensure patient-centred management protocols in small care homes where policies around staff and patients contacts are set for smaller scales [30].

We find no significant differences between for-profit and non-for-profit providers, although for-profit providers experienced the most excess deaths because they account for the majority of the market. A Canadian study showed for-profit status was not associated with the odds of an outbreak, although it was associated with the extent of an outbreak (number of cases and deaths) [8]. However, we find that branded care homes had greater odds of COVID-19-confirmed/suspected deaths and rates of excess deaths. Branded homes could have policies around staff and patient movement across facilities that could potentially aid the spread of infection [7], particularly in the earliest parts of the pandemic before policy caught up and/or in the face of staff absence.

## Conclusions

Specialist initiatives are needed for patients/staff/visitors to minimise the risk of initial infection in care homes. What prevention policies are optimal (e.g. polymerase chain reaction (PCR) testing, staff cohorting, visitor restrictions, hospital discharge policies, limiting visiting professionals, tracing staff) [31] requires further research and dialogue with operators and public health experts [28]. Their efficacy depends upon the care home setting in which they are implemented and the behavioural responses of residents and staff. Critically, any benefits from such policies would need to be weighed against costs and potential adverse outcomes, such as reduced quality-of-life or psychological well-being [28, 31].

There is an urgent need for accessible linked data of care home residents that could be used to inform service responses [32] and further research to explore the mechanisms hypothesised above in more detail. Evaluations of alternative interventions are also required. However, our results suggest that until this is possible, prioritising existing resources, such as testing and PPE equipment, to prevent initial infections in care homes is key to preventing large excess mortality.

## Supplementary Information

**Additional file 1: Appendix 1.** Construction of the analysis sample. This appendix includes three figures: **Figure A1-1.** Average death toll by day of the week in England. **Figure A1-2.** Variation in the weekly death toll across local authorities. **Figure A1-3.** Percentage of care homes experiencing COVID-19 deaths by care home size (number of beds). **Appendix 2.** Model selection and robustness to alternative specifications of seasonality. This appendix includes four Tables: **Table A2-1.** Results of the validation exercise for deaths in all care homes. **Table A2-2.** Results of the validation exercise for deaths in nursing care homes. **Table A2-3.** Results of the validation exercise for deaths in residential care homes. **Table A2-4.** Robustness of excess death estimations to alternative econometric models. **Appendix 3.** Predicted versus observed deaths, and estimated excess deaths over time (all care homes and by care home setting type), full observational period. This appendix includes two figures: **Figure A3-1.** Predicted versus observed deaths, and estimated excess deaths - All care homes in England. **Figure A3-2.** Predicted versus observed deaths, and estimated excess deaths over time by care home setting type. **Appendix 4.** Predicted versus observed deaths, and estimated excess deaths by whether care homes have experienced COVID-19 attributable deaths in the first 23 weeks of the pandemic. This appendix includes two figures: **Figure A4.1.** Predicted versus observed deaths, and estimated excess deaths over time – Nursing homes. **Figure A4.2.** Predicted versus observed deaths, and estimated excess deaths – Residential homes. **Appendix 5.** Multivariable adjusted odds ratios (sensitivity analysis). This appendix includes two Tables: **Table A5-1.** Multivariable adjusted odds ratios (95%CI) of experiencing COVID-19 confirmed/suspected deaths in the English care homes that provide services to older people/with dementia. **Table A5-2.** Multivariable adjusted odds ratios (95%CI) of an augmented model adjusted for whether care homes located in urban area (reference located in rural area) and whether in least and most deprived 20% of areas in England.

## Data Availability

Care home-level daily death notification data provided by the Care Quality Commission (CQC) and linked with CQC registers of active care homes in England. Neither the funders nor the collectors/curators/delivers of the data bear any responsibility for the analyses or interpretations presented here. The statistical code is available from the corresponding author.
